# Feasibility and Diagnostic Accuracy of Ultrastaging in the Detection of Micrometastases in Sentinel and Non-sentinel Lymph Nodes in Cervical Cancer: A Single-Center Retrospective Study With a Five-Year Follow-Up Period

**DOI:** 10.7759/cureus.61336

**Published:** 2024-05-29

**Authors:** Lavinia Balan, Elena Lavinia Rusu, Sebastian Ciurescu, Tomescu V Larisa, Cristina Secosa, Cristina Potre, Ligia Balulescu, Simona Brasoveanu, Madalina Alexandra Balica, Laurentiu Pirtea

**Affiliations:** 1 Department of Obstetrics and Gynecology, Victor Babes University of Medicine and Pharmacy, Timisoara, ROU; 2 Department of Internal Medicine, Victor Babes University of Medicine and Pharmacy, Timisoara, ROU; 3 Discipline of Infectious Diseases, Victor Babes University of Medicine and Pharmacy, Timisoara, ROU

**Keywords:** isolated tumor cells, micrometastasis, cervical lymph node metastasis, indocyanine green (icg), sentinel lymph node (sln), ultrastaging, uterine cervical cancer

## Abstract

Background: Cervical cancer is the fourth most common cause of malignant tumor-related deaths among women in developing nations. Cervical cancer has been estimated to cause 527.600 new cases and 265.700 deaths globally per year.

Objectives: This study aimed to evaluate patients with cervical cancer by ultrastaging all the lymph nodes (LN), sentinel LN (SLN) and non-SLN, to increase the sensitivity of the detection of LN metastases and the diagnostic accuracy in cervical cancer with a five-year follow-up.

Materials and methods: This is a retrospective study of 14 cervical cancer cases from 2017 to 2019 at the Municipal Emergency Clinical Hospital of Timisoara, Romania. The cases were selected based on their high risk of LN involvement but negative intraoperative pathologic LN. After re-evaluating all paraffin block biopsy samples from 29 cases, 14 cases were included in the study, which met all criteria for ultrastaging on surgical biopsy samples.

Results: Patients’ ages included in the study ranged from 43 to 70 years (median: 57.14 years). According to the International Federation of Gynecology and Obstetrics (FIGO) staging, the majority of the patients were in stage IB: seven cases (50%). The study revealed a positive correlation between patient age and FIGO staging, with Pearson’s correlation coefficient of 0.707 and a p-value of less than 0.05, indicating that older patients were more likely to be diagnosed with a higher FIGO stage. The mean follow-up was 34.5 months, and the median follow-up was 36 months (range: 6-60 months). We obtained 167 nodes, with a mean of 11.92 nodes/case. Twenty-one LN were found to be positive with the ultrastaging method. We detected 11 LN with macrometastases (MAC) (52.38%), seven with micrometastasis (MIC) (33.3%), and three with tumor cell islets (14.4%). That would be 13% of newly diagnosed ultrastaging cases as positive nodes. This ultrastaging method detected nodal MIC in eight (57.1%) out of the 14 patients, who initially tested negative for LN involvement using the routine Hematoxylin and Eosin (HE) method. The detection of micrometastases in these patients underscored the superior sensitivity of ultrastaging, which was further highlighted by the subsequent relapse of four (28.57%) out of these eight patients. The study also found no correlation between the FIGO standardization and the number of MIC found in these patients.

Conclusions: Predicting cervical LN metastasis (LNM) is crucial for improving survival rates and reducing recurrence. Very few small cohort studies used an ultrastaging method to assess non-SLNs; most of them only assessed SLNs. We showed in our study that the ultrastaging method, both in the case of SLN and non-SLN, is superior compared with H&E analysis, with a 13% rate of new positive nodule diagnosis. Metastatic involvement of non-SLN was found in over 50% of all cases (8/14) according to the ultrastaging method. Additionally, our study confirms that the sensitivity of SLN ultrastaging is high for the presence of both MIC and MAC in SLN pelvic LN. As a result, we feel that ultrastaging is the most effective method for SLN analysis in patients with early-stage cervical cancer, and bilateral detection is preferable, significantly reducing false-negative results. The routine use of SLN along with ultrastaging would lead to more accurate surgical staging and better oncological follow-up of cases.

## Introduction

Cervical cancer is the fourth most common cause of malignant tumor-related deaths among women in developing nations. Cervical cancer has been estimated to cause 527.600 new cases and 265.700 deaths globally per year [1].

Persistent infection with the sexually transmitted human papillomavirus (HPV) is the most frequent cause of cervical cancer. For women, particularly those under 35, HPV is responsible for 90-100% of occurrences of this malignity [2].

Cervical cancer is a malignant tumor of the cervix, divided into two types: adenocarcinoma (AC) and squamous cell carcinoma (SCC). SCC is more common and has a 70% occurrence rate. AC originates from glandular cells in the endocervix, while SCC originates from squamous cells in the transformation zone, where most tumors originate [2].

Treatment choices are heavily influenced by the status of pelvic lymph nodes (LN), which is a significant prognostic factor for cervical cancer. Regardless of the International Federation of Gynecology and Obstetrics (FIGO) stage, the five-year survival rate falls from 92% to 64% in the event of positive pelvic LN [2,3]. When cervical cancer is detected in its early stages (FIGO IA2, IB1, IIA), guidelines urge a pelvic lymphadenectomy to discover metastases and modify treatment. Pelvic lymphadenectomy is not indicated in stage 1A1 disease without lymphovascular space invasion due to the low incidence of LN metastases (LNM) (<1%) [3].

For many years, pelvic lymphadenectomy was the standard method for staging LN in the early stages of cervical cancer. A whole pelvic lymphadenectomy is linked to several side effects and morbidities, including lymphocele formation and lymphedema with longer operative time, and more intraoperative complications (bladder, bowel, nerves, and vessels) than sentinel LN (SLN) [4].

LNM, one of the most common forms of metastasis in cervical cancer, has a considerable influence on patient outcomes. Patients with cervical cancer with a positive LNM had a significantly worse five-year survival rate (35%-69%) compared to those with no metastases (91%). LNM has been identified as an independent risk factor influencing the prognosis of cervical cancer patients [1].

The SLN is the first site of tumor metastasis; thus, its pathology should reflect metastatic illness in other LNs in the basin. Lymphatic mapping and SLN biopsy may eliminate the need for a full regional lymphadenectomy in many cases [5]. Sentinel node mapping has been effective for gynecological malignancies [6]. Several multicenter studies have been published on sentinel node mapping in cervical cancer, including SENTICOL [7], AGO [8], and international cohort research [4,9].

Many studies have been conducted on detecting SLN with accurate and secure tracers, particularly for clinical applications. Since it can reveal the location of the local and regional LN in patients with cervical cancer, its detection lowers the morbidity rate. SLN identification does not always require a full lymphadenectomy; rather, an identified region might be treated [10]. Recently, Jewell et al. [11] published a study that used indocyanine green (ICG) in gynecologic cancers and concluded that the optimal bilateral mapping of ICG alone was 79% (156/197) and it was 77% (23/30) for ICG and blue dye (BD). That means ICG has a high bilateral detection rate and appears to offer an advantage over using BD alone [10,11].

The application of an ultrastaging methodology and tissue processing are crucial components of SLN adoption in cervical cancer. Research has demonstrated that 10%-15% more patients with micrometastasis (MIC) can be identified by SLN ultrastaging. Retrospective investigations indicate that MICs are associated with a comparable negative impact [12].

The technique of ultrastaging for treating cervical cancer has been incorporated into standard practice and is referenced in internationally recognized treatment guidelines, including the clinical practice guidelines of the European Society for Medical Oncology and the National Comprehensive Cancer Network. A variety of traces have been suggested for sentinel LN detection in an effort to permanently alter it and increase the method's effectiveness, which is measured by the rates of true positive and false negative detection rates. Technetium methylene blue (TMEB) and ICG are extensively used tracers that are frequently acknowledged. In addition, ICG is included in the guidelines stated above, indicating the method's widespread acceptance and usefulness. Recent experimental research has shown that ICG may have additional advantages for individuals with early-stage cervical cancer who are to undergo surgery [13].

The pathology protocol for SLN evaluation has not yet been defined; however, optimal bilateral detection of SLNs and the pathologic investigation of the SLN are intimately related to the procedure's sensitivity and specificity. It has been demonstrated that SLN ultrastaging increases the negative predictive value by up to 100% from 91%. If traditional pelvic lymphadenectomy is replaced with SLN biopsy alone, SLN ultrastaging needs to be part of the care [12].

There are still some unresolved issues, though, such as the SLN detection rate, which can be affected by the surgical technique, the amount and kind of tracers used, the correctness of the diagnosis, and the procedure's sensitivity and specificity [12,14]. The routine usage of an SLN biopsy in practice is influenced by all of these considerations [11].

This study aimed to evaluate by ultrastaging method all the LN, SLN and non-SLN, in patients with cervical cancer and high risk of LN involvement but a negative intraoperative pathologic LN assessment, to increase the sensitivity of the detection of LN metastases and the diagnostic accuracy in cervical cancer with five-year follow-up. Analysis of these non-SLN with ultrastasiging could reveal occult metastases, leading to underdiagnosis and undertreatment of patients, which could explain the relapse of the disease shortly.

## Materials and methods

Design of the study and ethics

The study was conducted according to the guidelines of the Declaration of Helsinki and approved by the Ethical Committee of the Municipal Emergency Clinical Hospital of Timisoara (number 81/12.12.2022). All women who participated in this study provided written informed consent. This study followed a retrospective design, and all patient data have been anonymized.

To evaluate the outcomes of the study, a comprehensive analysis of various parameters was conducted. These parameters included (i) demographic characteristics (age and medical history); (ii) surgery (type of surgery, SLN); and (iii) tumor characteristics (FIGO stage, histological diagnosis).

Selection of patients

Patients affected by cervical cancer who underwent radical hysterectomy and total pelvic lymphadenectomy in the Obstetrics-Gynecology Clinic of the Municipal Emergency Clinical Hospital of Timisoara from 2017 to 2019 were analyzed by two experienced anatomopathologists.

Only patients with a high risk of LN involvement but a negative intraoperative pathologic LN assessment were enrolled in the study. The following inclusion criteria were used: a) squamous carcinoma, adenocarcinoma, or cervical in situ carcinoma of the uterine cervix confirmed by histology; b) use of the SLN detection technique; c) no bulky or suspicious LNs on preoperative imaging; and d) planned surgical treatment, including LN staging. Only those patients with LN detection and a negative intraoperative pathologic LN evaluation were included. Patients without radical surgery were excluded. Similarly, patients with evidence of LNM on magnetic resonance imaging and/or computed tomography at their preoperative evaluation were not included, and patients in whom LNM were detected by routine histological examination were excluded.

Within the selected time frame, there were 148 patients diagnosed with cervical cancer in our hospital, and 100 received total hysterectomy and pelvic lymphadenectomy. Following the application of the inclusion criteria, 29 patients were selected to perform ultrastaging on surgical biopsy samples. After applying the exclusion criteria, 14 cases remained in the study that met all the criteria. Bioptic samples were processed following routine pathologic processing rules.

Surgery technique

A technique with ICG fluorescence was used for SLN detection either by laparoscopy or by laparotomy. A dose of 25 mg of ICG (ICG Pulsion, Pulsion Medical Systems SE, Feldkirchen, Germany) was dissolved in 20 mL of sterile aqua, leading to a concentration of 1.25 mg/mL. Directly before surgery, four doses of 1 mL each of this solution were then injected into four quadrants.

**Figure 1:**
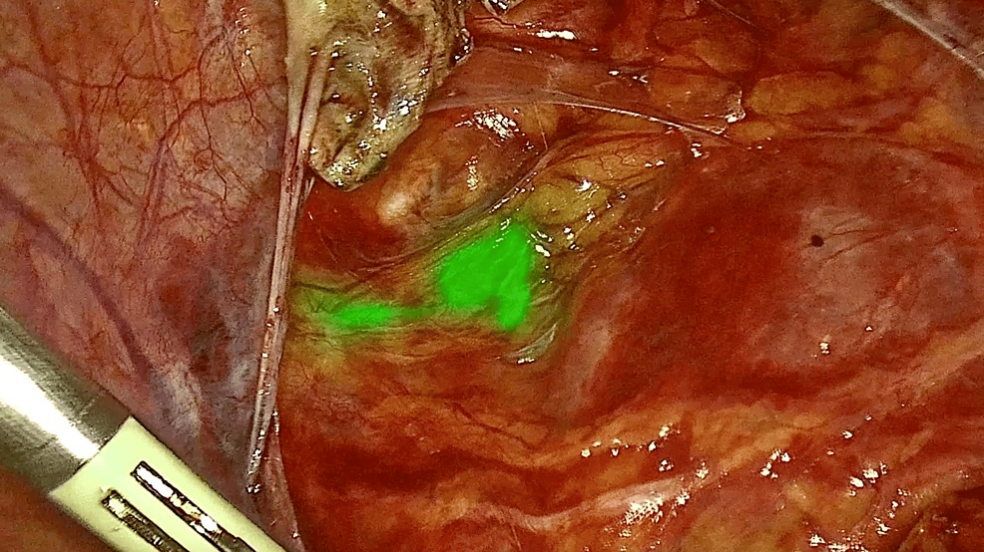
The sentinel diagnostic of an ilioobturatory node with ICG. ICG, indocyanine green fluorescence

The sentinel diagnostic and extirpation procedures were started a minimum of 10 minutes after the ICG injection (Figure 1). This time was used to install the pneumoperitoneum, the laparoscopic instruments, and the camera system. An endoscopic ICG system was used for ICG sentinel diagnostics (ICG system with D-Light P, Karl Storz, Tuttlingen, Germany) or to perform open surgery.

LN were removed from eight standard regions (external iliac left and right, obturator left and right, common iliac left and right, paraortic, and lateroartic). Patients with macrometastases (MAC) detected on intraoperative assessment were excluded from further analysis. All pelvic LNs, including SLNs and non-SLNs, were processed according to the pathological protocol for SLN ultrastaging.

Pathologic processing

**Figure 2:**
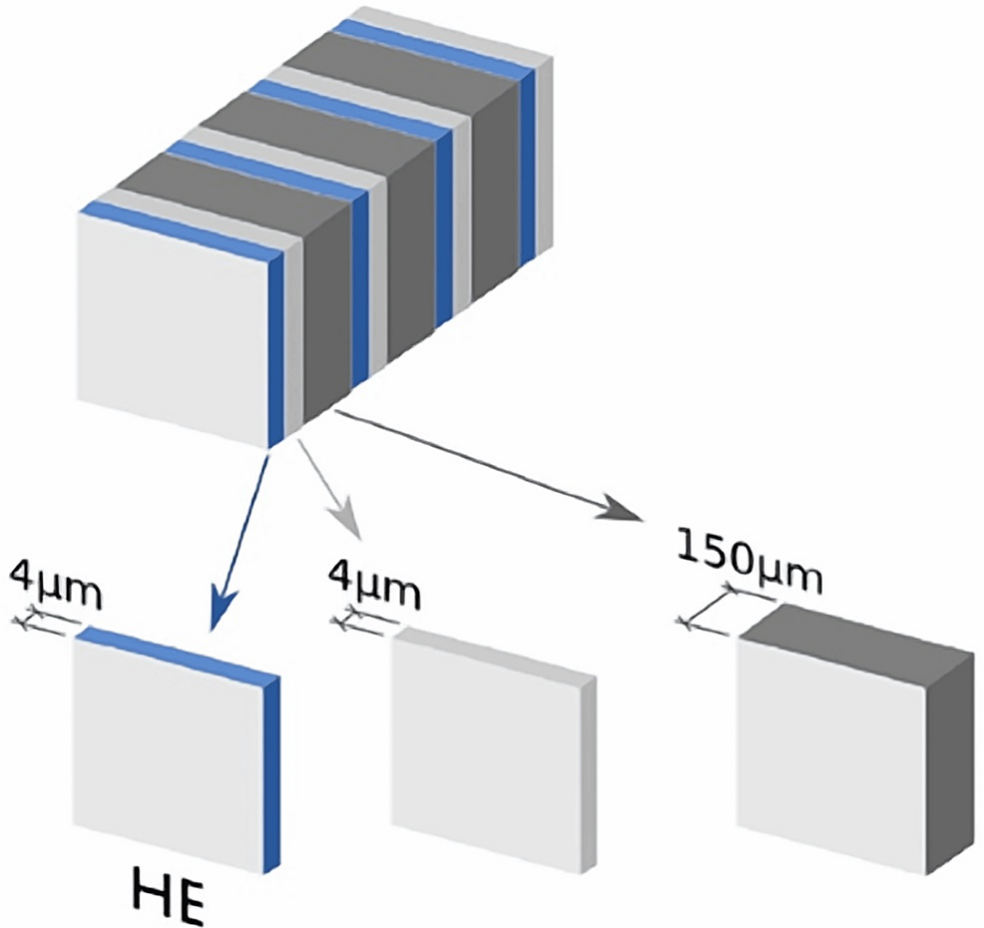
Protocol for pathologic processing of SLNs and all pelvic lymph nodes. SLNs, sentinel lymph nodes HE, hematoxylin-eosin staining

At the time of surgery, the LNs were cut along their longest axis. Primary processing of surgical biopsies SLNs, as well as all other non-SLNs, were fixed in 10% buffered formalin for 24-48 hours before being paraffin-embedded using all of the methods necessary to generate paraffin blocks formalin-fixed paraffin-embedded (FFPE). After fixation, all LNs were sliced into consecutive sections (4 μm thick) obtained in regular 150 μm intervals, which were cut from each paraffin block until no LN tissue was left (Figure 2) [15].

All specimens obtained from surgical procedures were processed and examined in a pathological anatomy laboratory specialized in histopathology.

LNs, defined as negative by HE routine pathology, were then analyzed by an ultrastaging technique. The presence of MAC, MIC, and isolated tumor cells (ITCS), was recorded and classified according to the TNM system. ITCs were defined as <0.2 mm, MIC as between 0.2 and 2 mm, and MAC as >2 mm.

Statistics

In this research, we employed a robust statistical approach to analyze the data collected from a comprehensive retrospective investigation of 100 patients who had undergone total hysterectomy with lymphadenectomy. Our statistical methodology was underpinned by several key techniques: we utilized chi-square tests to determine the statistical significance of our findings. This non-parametric test was instrumental in assessing the independence of two categorical variables, providing us with a p-value to gauge the likelihood that any observed difference between the variables occurred by chance. In addition to the chi-square tests, we conducted a post-hoc Bonferroni test. This test was used to control the family-wise error rate and reduce the chances of obtaining false-positive results when multiple pairwise tests were performed on a single set of data. To investigate any potential link between age and FIGO staging, we employed linear correlation. This technique allowed us to quantify the strength and direction of the relationship between these two continuous variables. Our statistical approach underscored the superior sensitivity of ultrastaging in detecting nodal micrometastasis, which was further highlighted by the subsequent relapse of four out of the eight patients who initially tested negative for LN involvement using the routine HE method.

## Results

The present study is a descriptive, retrospective study that includes a total of 14 patient cases. Two experienced anatomopathologists selected these cases by re-evaluating all the paraffin block biopsy samples microscopically from 29 cases diagnosed with cervical cancer and undergoing total hysterectomy with lymphadenectomy during the period selected for the study. After applying the exclusion criteria, 14 cases remained in the study that met all the criteria. Bioptic samples were processed following routine pathologic processing rules for ultrastaiging.

Patients’ ages ranged from 43 to 70 years (median: 57.14 years). Histology showed squamous cell carcinoma in 10 patients, adenocarcinoma in three, and microinvasive carcinoma in one. The selected patients were characterized by their menopausal status, with nine patients (64.3%) in menopause and five (55.6%) at fertile age. The International Federation of Gynecology and Obstetrics staging was from stage IA to stage IIIC. The highest proportion was in the following stages IB: seven cases (50%), IA: three cases (21.4%), IIIC: three cases (21.4%), and IIB: one case (7.1%). Eleven (78.6%) patients had vascular invasion. The mean follow-up was 34.5 months, and the median follow-up was 36 months (range: 6-60 months).

In four (28.6%) cases, a hysterectomy with classical lymphadenectomy was performed, and in 10 (71.4%) cases, a hysterectomy was performed with laparoscopic lymphadenectomy. By the laparoscopic route, we obtained a total of 133 LN, and by the classical route, 34 LN.

The characteristics of the 14 patients with clinically negative LNM are summarized in Table 1.

**Table 1:** Patient characteristics. The data are presented as n (%). FIGO, The International Federation of Gynecology and Obstetrics

Main Characteristics of the Study Population	No. (Percent) of Cases
Age (years)	
<50	3 (21.4%)
50-60	5 (37.5%)
>60	6 (42.9%)
Menopause	
Yes	9 (64.3%
No	5 (55.6%)
Stage (FIGO)	
IA	7 (50%)
IB (B1-B3)	3 (21.4%)
IIB	1 (7.1%)
IIIC	3 (21.4%)
Histology	
Adenocarcinoma	10 (71.4%)
Adenosquamous cell carcinoma	3 (30%)
Microinvasive carcinoma	1 (7.1%)
Grading	
G1	1 (7.14%)
G2	9 (62.29%)
G3	4 (17%)
LVI (Lymphovascular invasion)	
Yes	11 (78.6%)
No	3 (27.3%)
Procedure	
Classical radical hysterectomy	4 (28.6%)
Laparoscopic radical hysterectomy	10 (71.4%)
LN per case	
5-10	5 (35.7%)
10-15	7 (50%)
>15	2 (14.3%)

**Figure 3:**
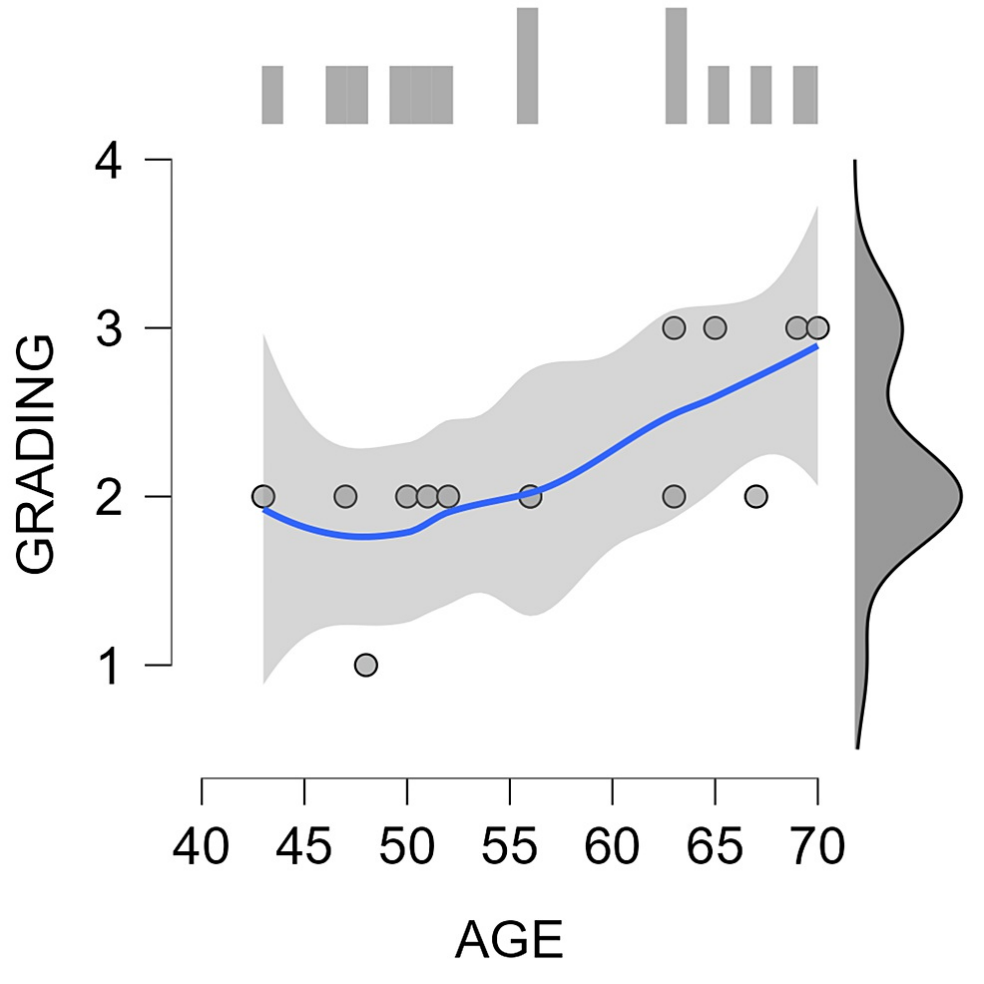
Correlation between patient age and FIGO staging. FIGO, The International Federation of Gynecology and Obstetrics

The study also revealed a positive correlation between patient age and FIGO staging, with Pearson’s correlation coefficient of 0.707 and a p-value of less than 0.05, indicating that older patients were more likely to be diagnosed with a higher FIGO stage (Table 2 and Figure 3).

**Table 2:** Correlation between patient age and FIGO staging. FIGO, The International Federation of Gynecology and Obstetrics

Pearson's Correlations			
Variable		Age	Grading
1. Age	Pearson's r	—	
	p-value	—	
2. Grading	Pearson's r	0.707	—
	p-value	0.005	—

The tumor, node, and metastasis (TNM) staging variations of the group are shown in Table 3.

**Table 3:** Frequencies for tumor, node, and metastasis (TNM) staging.

TNM	Frequency	Percent	Valid Percent	Cumulative Percent
pT0 N0 Mx	1	7.143	7.143	7.143
pT1a1 NX Mx	1	7.143	7.143	14.286
pT1b1 N0 Mx	5	35.714	35.714	50.000
pT1b1 N1 Mx	2	14.286	14.286	64.286
pT1b2 N0 Mx	2	14.286	14.286	78.571
pT2 N0 Mx	1	7.143	7.143	85.714

Out of 14 cases included in the study, three cases were detected in the sentinel LN by ICG. These cases were then continued with a complete pelvic lymphadenectomy. In 11 cases, no SLN was detected, and a complete lymphadenectomy was decided. In five cases, a lymphadenectomy was performed until the para-aortic level, and we founded two positive LN one with MIC, and one with MAC. For the patients with LSN, they had a sentinel node in one of the following two sites: the external iliac region (two nodes) and the obturator region (three nodes). 

**Figure 4:**
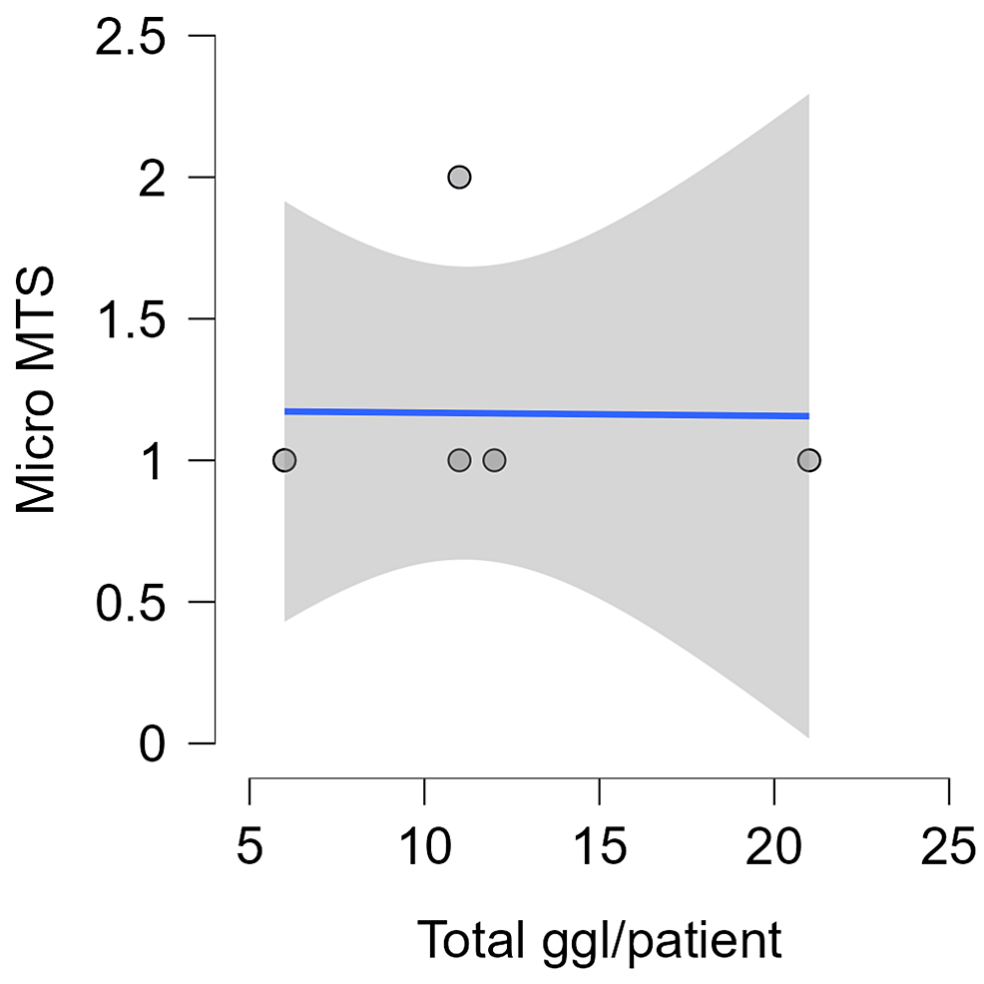
Total lymph nodes/patient - Micro MTS. MTS, metastasis; ggl, ganglion cardiacum (lymph nodes)

**Figure 5:**
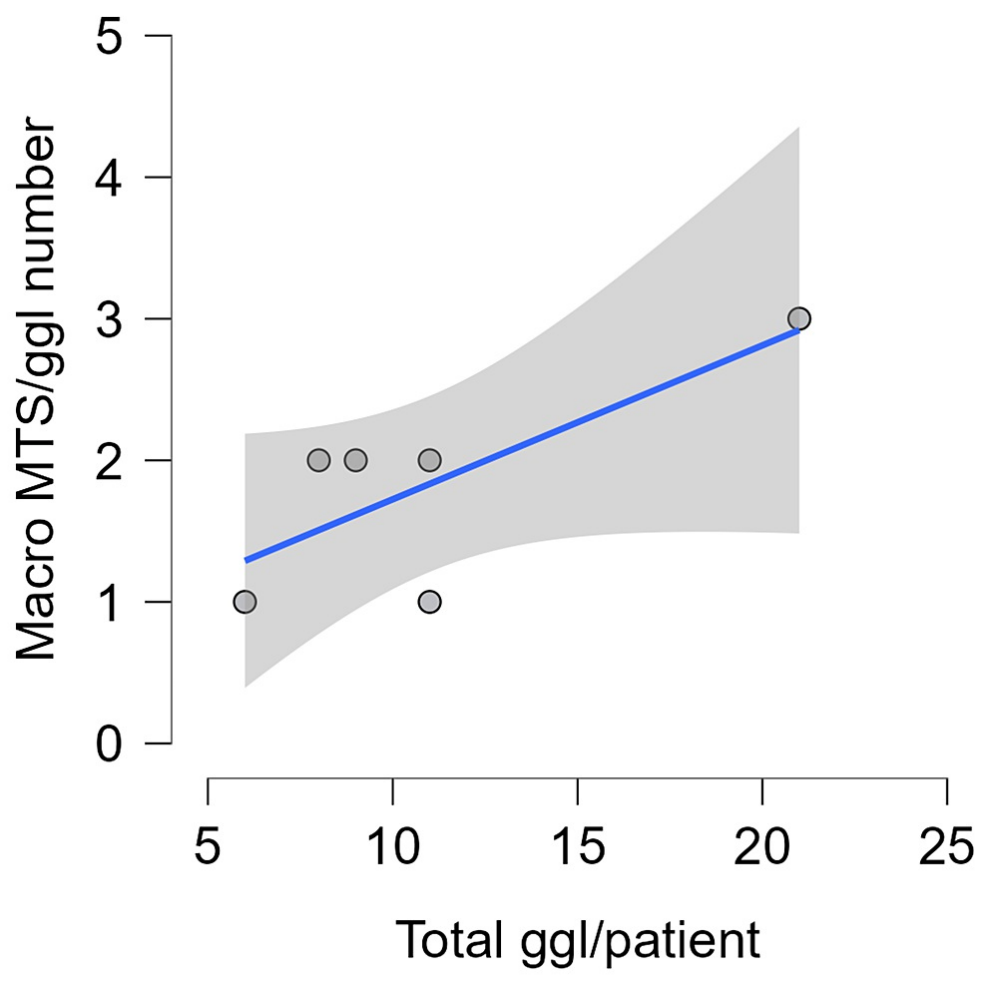
Total lymph nodes/patient - Macro MTS. MTS, metastasis; ggl, ganglion cardiacum (lymph nodes)

**Figure 6:**
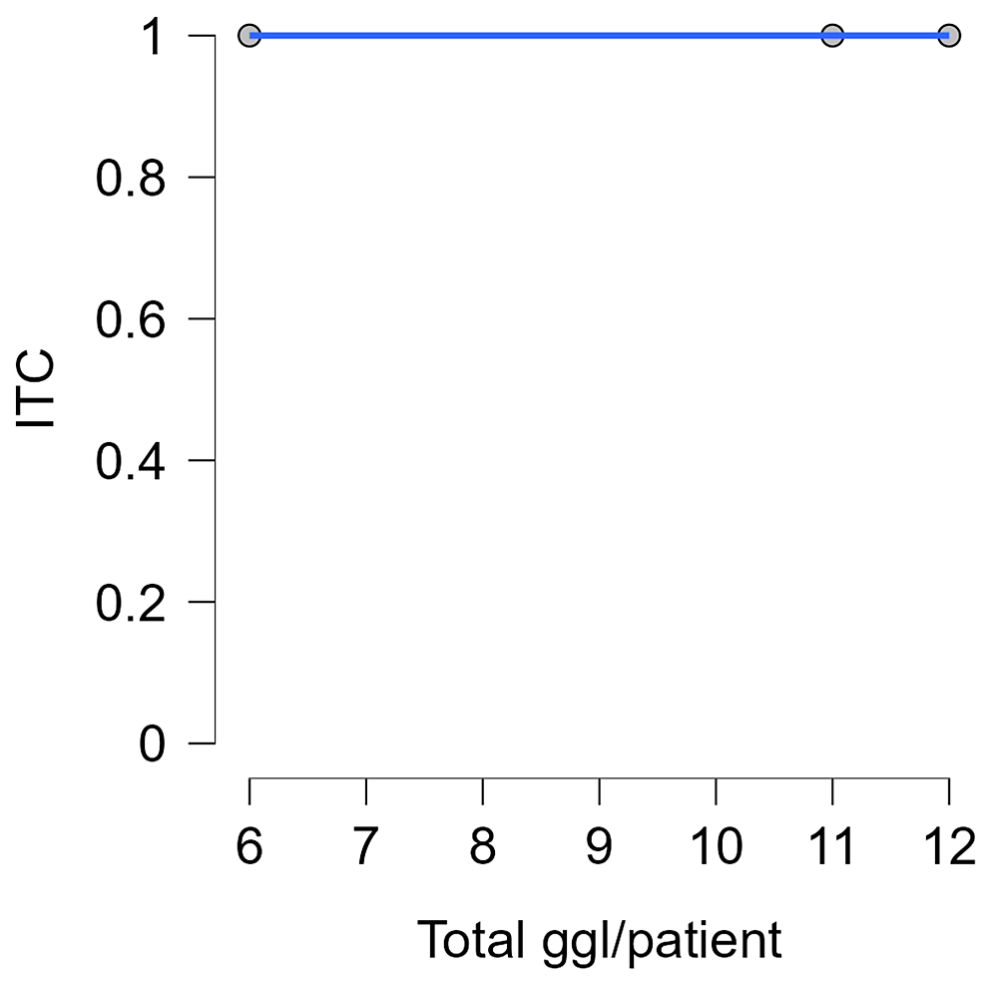
Total lymph nodes/patient - ITC. ITC, isolated tumor cells; ggl, ganglion cardiacum (lymph nodes)

Of the 167 nodes studied, with a mean of 11.92 nodes/case (range: 6-29 LN). Twenty one LN were found to be positive with the ultrastaging method. We detected 11 LN with MAC (52.38%), seven with MIC (33.3%), and three with tumor cell islets (14.4%). That would be 13% of newly diagnosed ultrastaging cases as positive nodes. The locations and number of LSN and nodes MIC detected are summarized in Table 4 and Figures 4-6.

**Table 4:** The location of LN and MIC. C, common iliac; MAC, macrometastases; MIC, micrometastasis; ITC, isolate tumor cell; R, right; L, left; O, obturator; I, iliac; Lao, latero- and para-aortic; +, positive; -, negative; SLN, sentinel lymph node; non-SLN, non-sentinel lymph node; LN, lymph node

Patient No.	Micrometastases			SLN No.		Non-SLN No.	
	SLN	Non-SLN No.	LN Basin	R	L	R	L
1	-	+MIC, ITC	C	-	-	7	5
2	-	+MAC	RO, LO, RI, LI	-	-	3	5
3	-	+MIC, ITC	C, Lao	-	-	1	1/4
4	-	+MAC	C, Lao	-	-	3	2/4
5	-	+MAC, MIC	LI, C, Lao	-	-	0	3/5/8
6	-	+MIC	C	-	-	8	3
7	-	+MAC, MIC	RO, LO, RI, LI	-	-	12	4
8	-	+MAC, MIC	RO, RI, LI	-	-	5	1
9	-	-	RO, LO, RI, LI	1	1	6	17
10	-	+MAC	LI, C, Lao	1	1	2	1/4/3
11	-	+ ITC	C, Lao	-	-	3	6/2
12	-	-	LO, RO, RI, LI	-	-	6	3
13	-	-	C	-	-	6	8
14	-	-	LO, RO, RI, LI	1	1	4	7

This ultrastaging method detecting nodal MIC in eight (57.1%) out of the 14 patients, who initially tested negative for LN involvement using the routine HE method. The detection of MIC in these patients underscored the superior sensitivity of ultrastaging, which was further highlighted by the subsequent relapse of four (28.57%) out of these eight patients at varying intervals - five years, three years, two years, and six months, respectively. Only one of the cases with relapse remained negative, and after ultrastaging, no MIC were found here. In the other three cases, MIC and tumor islets were found, and in one case, MAC (this is also the one that had the fastest recurrence at six months, with the patient's death at two and a half years). Two cases were classified as stage IA, one as stage IB, and one as stage IIB. 

These four cases with relapse highlighted the superior sensitivity of the ultrastaging method. These patients would not have been diagnosed in a timely manner using conventional diagnostic methods, emphasizing the critical role of ultrastaging in early detection and intervention.

There were no cases of false positives diagnosed by the classical reading method as compared to the ultrasound method.

For the three cases where an SLN was obtained using the ultrasound method, the same results were obtained as in the classical routine HE method. This demonstrates the specificity of the sentinel LN. In those with SLN, the rest of the node stations were checked by ultrastaging and remained negative.

The significant statistical value (p-value <0.05) suggests that the benefits of using the ultrastaging method outweigh these limitations, underscoring its potential as a valuable tool in the early detection and management of cervical cancer.

A P value of <0.05 is statistically significant, suggesting that using ultrastaging method would be beneficial compared to standard tests.

We also employed the use of T tests and F tests to discern patterns and relationships within the data. In this study, these tests were meticulously applied to examine the potential associations between the age of patients or their FIGO staging and the presence of MIC. However, the results of these tests did not yield any significant differences (p-value >0.05, Table 5).

**Table 5:** Correlation between FIGO staging, age, and presence of micrometastasis. FIGO, The International Federation of Gynecology and Obstetrics

	P-values of T and F Tests Applied			
	FIGO Staging/presence of micrometastasis		Patient's age/presence of micrometastasis	
F Test	F	0.649275	F	1.113212
	P(F<=f) one-tail	0.327893	P(F<=f) one-tail	0.431939
T Test Unequal Variances	t Stat	0.673152	P(T<=t) one-tail	0.227042
	P(T<=t) one-tail	0.256803	t Critical one-tail	1.795885

This lack of statistical significance implies that neither the age of the patients nor their FIGO staging could be reliably associated with the presence of MIC. In other words, these factors may not serve as reliable predictors for the occurrence of MIC in this specific patient population. Furthermore, the study found no correlation between FIGO staging and the number of MIC found in these patients. This indicates that the stage of the disease, as determined by the FIGO staging, does not necessarily correspond to the number of MIC. These findings underscore the complexity of the disease and the challenges in predicting its progression. They also highlight the need for further research and the development of more sophisticated predictive models that can take into account a broader range of factors. Despite the lack of significant findings in this study, the research provides valuable insights and contributes to the ongoing efforts to improve the early detection and treatment of this disease.

## Discussion

Predicting cervical LNM is crucial for improving survival rates and reducing recurrence. Early cervical cancer's LN status impacts prognosis and treatment choices. Pathological examination is the gold standard for diagnosing LNM, but it can be slow and ineffective. Systematic LN dissection increases surgery difficulty and the risk of organ damage, especially for patients with long-term complications. Preoperative attempts to develop predictive models for LNM risk factors using test results have helped some patients avoid surgery and radiotherapy-induced damage [1]. The effectiveness of lymphadenectomy in early-stage cervical cancer patients remains controversial, with an incidence rate of 7%-20% [1,16,17].

Sentinel nodes affected by uterine cervical cancer are often located in typical pelvic locations, such as the external iliac, obturator, and internal iliac basins. Outside the pelvis, common iliac sentinel nodes are most often discovered, but para-aortic, presacral, inguinal, and hypogastric nodes are also sometimes seen. The unusual location of sentinel nodes can have clinical consequences and may be overlooked by normal surgical techniques, leading to undertreatment in patients with metastases impacting these nodes [4].

LNM occur in up to 27% of early-stage cervical cancer patients, increasing the risk of needless pelvic LN dissection (PLND) and considerable morbidity. An SLN operation may be an appealing option to regular PLND since it accurately detects metastases and can be used as a substitute for complete LN dissection in the event of a negative SLN [18,19]. However, the procedure's success is determined by its detection rate and diagnostic accuracy. There is a continuing dispute over whether detection should be bilateral or per pelvic side, with a single or double tracer, and if the SLN should be ultrastaged. Recent evaluations have demonstrated that bilateral SLN detection and ultrastaging are safer and more effective than unilateral detection, frozen section, and HE analysis [3,4,20].

The ultrastaging protocol has been shown to improve the sensitivity of identifying LN involvement. Although worldwide associations encourage the treatment, it has not yet been standardized, and there are currently no standards available for the ultrastaging method [11].

Very few small-cohort studies used an ultrastaging method to assess non-SLNs; most of them only assessed SLNs. In one systematic review by Tax et al., which evaluated 47 studies (4,130 patients with early-stage cervical cancer IA2, IB1, and IIA primary tumor size <40 mm) with bilateral negative SLNs after ultrastaging, they concluded that an SLN procedure may replace a full pelvic LN dissection. This procedure reduces the PLND overtreatment rate from 80% to 10%, with an acceptable risk of occult metastases of only 0.08% [3].

In a similar study, with a group of 17 patients with early-stage but high-risk cervical cancer, it was discovered in SLN through the final ultrastaging of isolated tumor cells in three cases, MIC in four cases, and MAC in one case, despite negative intraoperative SLN evaluation. Pathologic ultrastaging was used to process all extracted LNs, including pelvic non-SLN and SLN. They discovered that pelvic LNs had 100% sensitivity for both MAC and MIC in SLN ultrastaging. Not a single instance of MIC in non-SLN or negative SLN was discovered [15].

We had similar results for the specificity of SLN, but in our study, we showed that the ultrastaging method, both in the case of SLN and non-SLN, is superior compared with HE analysis with a 13% rate of new positive nodule diagnosis. Most cases had MAC (11 nodules) and MIC (seven nodules), and only three nodules with isolated tumor cells were detected. Above all, we did not have any cases of false-positive diagnoses.

Ultrastaging provided the best diagnosis accuracy, revealing SLN metastases in 80% (75/94) of early-stage cervical cancer patients whose SLN was considered negative on frozen section analysis and/or HE stain examination. Recent studies have also demonstrated the therapeutic importance of eliminating low-volume disease, including isolated tumor cells, which are primarily observed using ultrastaging [9]. As a result, we feel that ultrastaging is the most effective method for SLN analysis in patients with early-stage cervical cancer. However, bilateral detection is preferable because it achieves the maximum diagnostic accuracy by significantly reducing false-negative findings. Therefore, bilateral detection should be a necessity. Tax et al. recommend doing a thorough pelvic LN dissection whenever the SLN is not bilaterally found [3].

Using a double tracer approach for ultrastaging, early-stage cervical cancer patients with a primary tumor size of <40 mm can obtain a high bilateral detection rate and a low false-negative rate. When the following criteria were satisfied as preliminaries, diagnostic accuracy was the greatest, with an estimated sensitivity of 99.6% (95% CI: 98-100%) and 99.9% NPV: bilateral SLN detection, no questionable LN on pre-operative imaging or during surgery, and a main tumor diameter of 40 mm. These parameters lower the residual probability for occult metastases to 0.08% (1/1257). In comparison, in melanoma, breast, and vulvar cancer, a residual risk of 3%, 0-10%, and 2%, respectively, for occult metastasis has been regarded as an appropriate cut-off point to discontinue comprehensive LN dissection [3,21-23].

The most significant component of any SLN method is diagnostic accuracy. To be a safe substitute for routine LN dissection, it should ideally be as high as feasible. As it indicates the percentage that an SLN technique could be able to prevent a pelvic lymphadenectomy, the detection rate should also be as high as possible. However, there should never be a situation where a higher detection rate results in lower diagnosis accuracy [15].

The false negative of SLN staging is a crucial factor for patient safety when it comes to the idea of substituting pelvic lymphadenectomy with SLN biopsy alone. For SLN, a negative SLN with a positive non-SLN is generally considered false negativity. Certain studies only classify false-negative SLNs when the mapped and histologically negative SLN are on the same side as the positive non-SLN. This strategy appears to be justified because contralateral pelvic lymphadenectomy is done on patients whose SLN has been detected unilaterally. On the other hand, certain studies label false-negative SLNs even in instances where patients had contralateral non-SLN positive and unilateral SLN detection. There were 127 studies with data available for false negativity, and the false-negative ratio was 2.4% (137 out of 5,734 patients). When these data were stratified according to the contralateral positive of non-SLNs and unilateral SLN identification, however, 39.5% (32 of 81 patients from 28 of 127 trials) of erroneous negativity was associated with this situation [12].

In our study, despite a negative intraoperative result, metastatic involvement of non-SLN was found in over 50% of all cases (8/14) according to the ultrastaging method. Additionally, our study confirms that the sensitivity of SLN ultrastaging is high for the presence of both MIC and MAC in SLN pelvic LN. Without the use of ultrastaging, the eight patients with MIC would not have been diagnosed in time, highlighting the method’s critical role in improving patient outcomes.

Radical surgery followed by adjuvant chemoradiation has the highest treatment complication rate, as each therapy induces multiple types of negative effects. As a result, new worldwide guidelines promote the intraoperative triage of patients based on LN evaluation. A radical uterine surgical procedure can be avoided if LN involvement is assessed during surgery, and the patient is referred for definitive chemoradiation [12].

The SENTICOL I study found that a bilateral negative SLN predicts LN involvement in remaining pelvic LN [7], while a positive SLN does not predict the status of downstream LN. Current guidelines and the ABRAX study suggest that metastatic SLN diagnosed at frozen section analysis should be completed with an additional paraaortic lymphadenectomy for nodal staging before definitive chemo-radiation [24].

These findings emphasize the critical role of ultrastaging in early detection and intervention and the importance of rigorous statistical analysis in medical research. Our statistical methodology provided a rigorous framework for analyzing our data, leading to significant insights into the effectiveness of ultrastaging in the early detection of nodal MIC. This approach, combined with our meticulous selection process and standard pathological processing protocols, underscores the robustness and reliability of our research findings.

The five-year overall survival rate for early-stage cervical cancer is greater than 90% [25]. LNM significantly reduces patient survival [26]. According to Hosaka et al., in patients with stage IB-IIB cervical cancer, the five-year overall survival (OS) rate was 94.8% in those without LNM [27]. Patients with LNM had a five-year survival rate of 62%. The prognosis was proportional to the number of metastatic LN [1]. In our study, the five-year overall survival rate was 92.9%, and the relapse rate of the disease was 28.57%.

The nodal status could influence the decision for the administration of adjuvant therapies, such as chemotherapy, radiation, or both. In conclusion, complete surgical staging with LN sampling is recommended for all women with high-risk cervical cancer [28].

Limitations

The following are the limitations of the study: (1) The main limitation of this study is that we have limited data to compare our results with those from other research centers involved in the study of non-SLN ultrastaging; (2) The second limitation of this study is the small size of the cohort; only 14 out of 100 cases met all the criteria. Only patients with a high risk of LN involvement but a negative intraoperative pathologic LN evaluation were enrolled in the study; (3) Additionally, the small size of the cohort is due to the availability of the data from patient records, its single-center design, and its financial cost. Data acquired from a single site may not be representative of the entire population, as patient background characteristics might vary greatly throughout the world. Given that the patient's addressability has increased at our hospital for cervical cancer detection and treatment, we will perform a comparison study on a larger number of patients in the future.

Despite the clear benefits of ultrastaging, it is important to acknowledge its limitations, including high cost, time-consuming procedures, and the need for specialized equipment and training. They also highlight the need for further research and the development of more sophisticated predictive models that can take into account a broader range of factors.

## Conclusions

Predicting cervical LNM is crucial for improving survival rates and reducing recurrence. Very few small cohort studies used an ultrastaging method to assess non-SLNs; most of them only assessed SLNs. We had similar results for the specificity of SLN, but in our study, we showed that the ultrastaging method, both in the case of SLN and non-SLN, is superior compared with HE analysis with a 13% rate of new positive nodule diagnosis. Metastatic involvement of non-SLN was found in over 50% of all cases (8/14) according to the ultrastaging method. Additionally, our study confirms that the sensitivity of SLN ultrastaging is high for the presence of both MIC and MAC in SLN pelvic LN.

As a result, we feel that ultrastaging is the most effective method for SLN analysis in patients with early-stage cervical cancer, and bilateral detection is preferable, significantly reducing false-negative results. Routine use of SLN along with ultrastaging would lead to more accurate surgical staging and better oncological follow-up of cases.
